# Age-Related Differences in Head Impact during Experimentally Induced Sideways Falls

**DOI:** 10.1155/2019/6804614

**Published:** 2019-04-22

**Authors:** Tyler A. Wood, Yaejin Moon, Ruopeng Sun, Alka Bishnoi, Jacob J. Sosnoff

**Affiliations:** Department of Kinesiology and Community Health, University of Illinois at Urbana-Champaign, 906 S. Goodwin Avenue, Urbana, IL 61801, USA

## Abstract

**Purpose:**

To examine head impact incidence and head acceleration during experimentally induced falls as a function of age.

**Methods:**

15 young adults (21.2±2.7) and 10 older adults (61.9±4.3 years) underwent 6 experimentally induced sideways falls. Participants fell sideways onto a 20cm crash pad. The number of head impacts was tabulated from video recordings and head acceleration was calculated from motion capture data. A total of 147 falls were analyzed.

**Results:**

The young group underwent 88 falls, in which 11.4% resulted in head impact. The older group underwent 59 falls, in which 34.5% resulted in head impact. A proportion analysis revealed older adults had a significantly greater proportion of head impacts than young adults (*X*^2^(1) = 11.445, p = 0.001). A two-way ANOVA only revealed a main effect of head impact on acceleration (*F*(1,142) = 54.342, p<0.001).

**Conclusion:**

The older adults experienced a greater proportion of head impacts during sideways falls. Head impact resulted in greater head acceleration compared to no head impact. Collectively, this data highlights the possibility that age-related neuromuscular changes to head control may result in elevated risk of fall-related TBIs. Future research examining mechanisms underlying increases in fall-related head impact is warranted.

## 1. Introduction

Traumatic brain injuries (TBIs) result from damaging forces being applied to the brain [1]. TBIs are a major cause of morbidity and mortality in older adults. Upwards of 80% of TBIs stem from head impact during a fall [2, 3]. As a result of fall-related TBIs, older adults experience extended hospital stays and a ~10% fatality rate [2]. The adverse consequences of fall-related TBI have led to the call for research focusing on underlying mechanisms [1].

Mechanisms contributing to the high prevalence of fall-related TBIs in older adults have traditionally focused on changes in tissue properties (i.e., bone-mineral density) and use of anticoagulants (increasing likelihood of hemorrhage) [3, 4]. Another possibility that has received less scientific scrutiny is that older adults may strike their head differently than young adults due to age-related changes in the neuromuscular system (declines in strength, flexibility, reaction time, etc.). From a biomechanical perspective, a fall-related injury occurs when the tissue experiences forces greater than its ultimate strength [5]. Although it is known that older adults, especially fallers, have deficits in protective movements during falls [6, 7], there is limited information concerning head impact. Understanding head impacts during falls may identify novel strategies to reduce fall-related head injury.

It is possible that older adults suffer a greater number of fall-related TBIs because they experience a greater number of head impacts during falls and head impact results in greater head acceleration. Consistent with this view, the sports medicine literature has illustrated that the risk of mild TBIs increases with greater head acceleration at impact in young adults [8–11]. Yet, there is limited information concerning head impacts and acceleration during falls. Thus, this current investigation sought to examine kinematics of the head during an experimentally induced sideway fall in healthy young and older adults. Sideway falls were examined since they have an increased risk for fall-related TBI [4]. It was hypothesized that older adults would impact their head more frequently than young adults and that head impact would result in greater head acceleration.

## 2. Methods

### 2.1. Participants

This study involves a secondary analysis of baseline data from a recently completed pilot, randomized controlled trial of a safe landing training study (NCT# 0017577)[12]. 15 young adults (5 females) and 10 older adults (3 females) participated in the experimental fall paradigm. All procedures were approved by the University of Illinois at Urbana-Champaign institutional review board. All participants provided written informed consent prior to taking part in the investigation.

The inclusion and exclusion criteria were designed to include healthy individuals who are capable of safely undergoing the procedures. The inclusion criteria included age between 18 and 30 years and 55 and 75 years, having body mass index (BMI) between 18.5 and 34.9 kg/m^2^, adequate bone mass index (t score>-1.0), right hand being dominant, being capable of performing 5-time sit to stand test within 10 seconds, and Montreal Cognitive Assessment (MoCA) score over 26. Exclusion criteria included history of fracture, stroke, neuromuscular disease, osteoporosis, tumbling or gymnastics training, taking anticoagulants, or being pregnant.

### 2.2. Study Procedures

After completing the informed consent process, participants completed the following assessments to insure they met inclusion/exclusion criteria before taking part in the falling procedures. First, participants had their height and weight quantified to calculate BMI. Next, participants had their bone mineral density measured with DEXA bone densitometer (Hologic QDR 4500, Hologic Inc, Waltham, MA). Participants completed the 5-time sit to stand test [13], the MoCA [14], and the short form of the physiological profile assessment (PPA) [15]. The short form of the PPA consists of 5 tests relating to visual edge contrast sensitivity, lower limb proprioception, finger reaction time, knee extension strength, and balance on a foam pad. Results of the individual tests are compared to age-matched norms to obtain a fall risk score [15].

Prior to the falling experiment, participants completed a 10-minute stretching session to reduce the risk of injury. Participants were also equipped with protective gear including a light-weight foam martial arts helmet and wrist guard.

**Figure 1:**
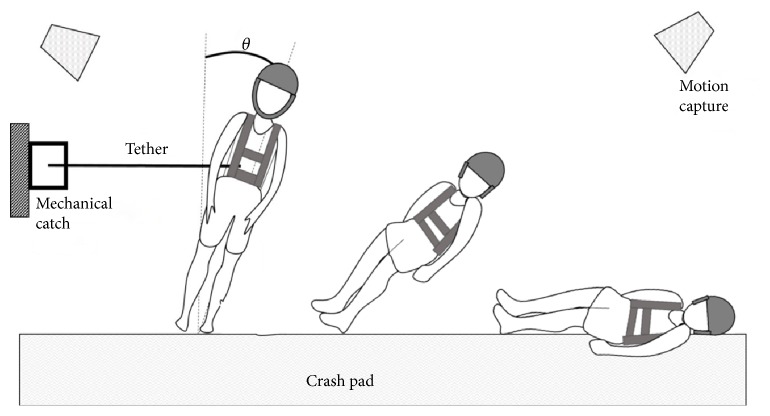
Experimental setup of a falling simulation. Image adapted from Moon 2018 [16].

During the experiments, participants were made to fall sideways onto a 183 cm x 122 cm x 20 cm thick crash pad (Asana Drag Pad, Asana Climbing & MFG, Boise ID, USA) by being released from a 10° side-way lean by means of an inextensible tether (Figure 1).

The 10° leaning angle is based on previous research, which exceeds the capacity of participants to recover from falling by taking a single step [17]. The tether was released via a mechanical catch (a snap shackle). Participants were unable to predict the time of release as a time delay between 3-8 seconds was randomly assigned to increase unexpectedness. Prior to tether release, the subjects were informed to look forward and keep the hips and knees extended. Participants were instructed to “land on the mat in a way that feels comfortable for you.” As hip impact was the main interest of the primary study, it was emphasized to land on the hip first by restraining from landing on the hand first, taking a step, or kneeling. The test consisted of 3 right-side falls and 3 left-side falls, for a total of 6 falls. Order of the direction of falls was counterbalanced.

### 2.3. Data Collection and Processing

Video of falls were recorded using an iPhone (Apple Inc. Cupertino, CA, USA) in the frontal plane at 60 Hz. TAW and two trained research assistants reviewed the video of each fall to classify the presence of head impact utilizing standardized procedures. Head impact was indicated if each of the following criteria were met: (1) head made contact with the ground (i.e., mat), (2) the mat deformed as a result of the head impact, and (3) the head rebounded from the mat before coming to rest. Any disagreement on impact classification was resolved through discussion.

A ten-camera motion capture system (VICON, Oxford Metrics, Oxford, England) was used to collect kinematic data of the head. Five reflective markers were attached on the anterior, posterior, bilateral, and superior parts of the helmet. The motion capture system tracked the 3D coordinates of the reflective markers at a sample rate of 100 Hz. The 3D coordinates of the markers were entered into a VICON Nexus software (VICON, Oxford Metrics, Oxford, England). A representative marker on the head was selected for further analysis as markers were occluded depending on the body configuration at impact. Since there was sudden change of trajectory of markers at impact, conventional gap filling methods for missing markers have a high likelihood of being inaccurate [18]. Therefore, to maximize accuracy, a representative marker was selected based on the following two criteria: (1) the marker did not have missing frames at the impact phase and (2) the marker was the closest to the impact point. Then, the VICON Nexus software calculated velocity data of the representative marker. We used a custom MATLAB script to filter the head velocity data using a low-pass-filtered with a fourth-order Butterworth filter with a cutoff frequency of 10Hz [19]. Then, head acceleration was computed by numerical differentiation of head velocity and subsequently low-pass-filtered with a fourth-order Butterworth filter with a cutoff frequency of 20Hz. Maximum head acceleration was calculated as a maximum value of head vertical acceleration. We neglected horizontal components of the parameters since it has been shown that they have relatively little effect on risk for injury during a fall [20].

### 2.4. Statistical Analysis

Statistical analysis was performed using SPSS for Windows, version 25.0 (IBM, Inc., Chicago, IL). Descriptive statistics were calculated for participant demographic information and independent sample t-tests were used to examine group differences. A chi-square test was used to examine whether there were differences in the proportion of head impacts between the young and older group. A two-way (age group by impact) analysis of variance (ANOVA) was used to examine acceleration differences between head impact and no head impact in the young and older group. Finally, a two-way (age group by impact) ANOVA was used to examine fall direction differences between head impact and no head impact in the young and old group.

## 3. Results

A total of 25 participants (15 young and 10 old) underwent the study procedures.  Table 1 displays the participants' demographic information. Overall, there were minimal differences between the young and older adults in demographic and performance metrics.

Table 2 displays the results of the subcomponents of the PPA and fall risk score. Older adults were found to have greater postural sway and worse edge contrast sensitivity than the young adults; yet, these age-related differences were still within healthy ranges [21]. There were no significant differences in simple reaction time, knee extensor strength, or lower limb proprioception.

Data from a total of 147 falls were recorded and analyzed. It was not possible to analyze three falls (2 young, 1 older) due to video loss. The young group experienced 90 falls, of which 88 were analyzed. Of these 88 falls, 11.4% (10 falls) resulted in head impact. Of the 15 participants in the young group, 6 participants experienced head impacts with the number of head impacts ranging from 1 to 3 per participant. The older group experienced a total of 60 falls, of which 59 were analyzed. Of the 59 falls, 34.5% (20 falls) resulted in head impact. Of the 10 participants, 7 participants experienced head impacts with the number of head impacts ranging from 1 to 5 per participant. Statistical analysis revealed that the older group experienced a significantly greater proportion of head impact than the young group (*X*^2^(1) = 11.445, p=0.001).

**Figure 2:**
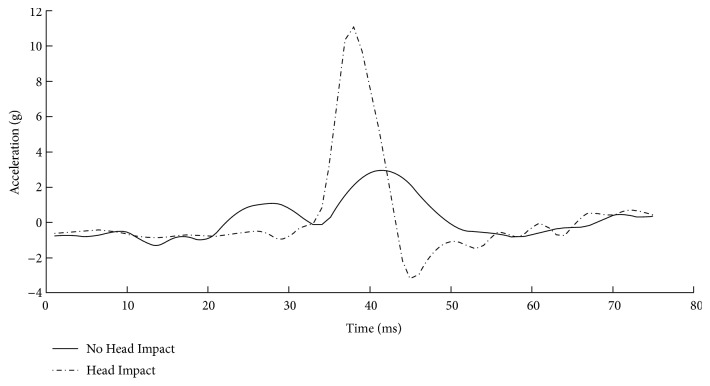
Head acceleration profiles for a 64-year-old male with no head impact and a 63-year-old male with no head impact.

**Figure 3:**
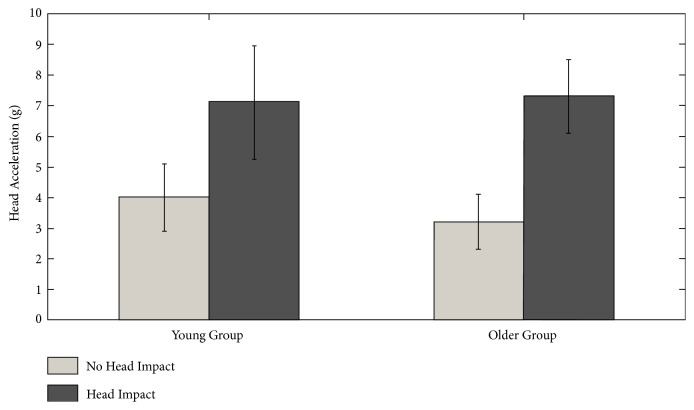
Head acceleration as a function of age group and impact.

Figure 2 displays a representative example of head acceleration profiles for a 64-year-old male with no head impact and a 63-year-old male with head impact. It is clear in the figure that falls with head impact have a greater head acceleration. Statistical analysis confirmed this observation. The two-way ANOVA revealed a main effect of head impact on acceleration (*F*(1,142) = 54.342, p<0.001). No effect of age (*F*(1,142) = 0.352, p=0.554) or an interaction between age and impact was observed (*F*(1,142) = 1.236, p=0.268).  Figure 3 displays head acceleration as a function of impact and age group. A two-way ANOVA revealed no effect of fall direction on head impact in either group (F(1,142) = 0.873, p=0.456).

## 4. Discussion

Fall-related TBIs in older adults are a major health concern [2, 3]. Previous investigations of fall-related TBIs have focused on tissue properties and use of anticoagulants as risk factors [3, 4]. This is the first investigation to examine age-related differences in head impact and quantify head acceleration during experimentally induced falls. The novel observation of this investigation was that healthy older adults had a greater proportion of head impacts than young adults. It was also noted that head acceleration was significantly greater with head impact than no head impact. These observations have significant importance considering the health implications of fall-related TBI in older adults.

There are multiple mechanisms that may contribute to the elevated proportion of head impacts in the older group. For instance, Yang and colleagues reported that older adults in long-term care with mild cognitive impairment, visual impairment and who are females were more likely to suffer a head impact during falls [22]. Although the older adult group in this investigation had lower edge contrast sensitivity than the young group, it is unlikely that it is related to higher impacts observed in the current investigation. It is important to note that while the edge contrast sensitivity of this group was lower than young adults, the score is above the normative value [21]. Additionally, Yang and colleagues did video analysis of real world falls while the current investigation utilized a simulated fall paradigm.

Another possible mechanism that may be contributing to head impact is age-related neuromuscular changes to the neck musculature. The neck is responsible for control of the head and age-related declines in neck strength and function may result in an inability to control the head during a fall [23, 24]. Neck strength decreases 35%-45% between the ages of 20 and 60 years [25]. Furthermore, it has been shown that the muscle fibers of the sternocleidomastoid (SCM), the main agonist of neck flexion and lateral flexion, remodel with age. In young adults, the SCM has similar amounts of Type I and Type IIa muscle fiber [26–29]. Yet with advanced age, the SCM takes on a slower muscle phenotype with a decreased area of fast twitch fibers (Type II) and increase in the number of slow twitch fibers (Type I) [26–29]. Collectively, these age-related changes to muscle strength and muscle fibers are likely to result in an inability of older adults to stabilize their head during a fall and experience head impacts. This speculation is consistent with sports medicine literature that highlights that lower neck muscle strength and slower muscle activation are risk factors for increased head acceleration and greater impact forces [30–32].

It is important to highlight the older group in the current investigation was relatively young (average age = 62 years of age) and physically fit. There were no age group differences in isometric knee extension strength or 5-time sit to stand test. The lack of strength differences between young and old is inconsistent with the possibility that age-related muscle strength changes contribute to head impact. It is possible that the rate of strength decline in neck musculature is greater than in lower limb musculature. Ultimately the mechanism(s) that contribute to increased head impact in older adults is unclear.

It was also observed that head impact resulted in greater head acceleration. This is concerning given that the sports medicine literature has consistently shown that increased head acceleration at impact is a risk factor for mild TBIs [8–11]. Head impact reconstructions from sporting events have estimated that the acceleration threshold for a mild TBI is 70*g* to 75*g* [8–11]. However, the acceleration threshold for fall-related TBI in older adults is not well understood. This observation may suggest that higher acceleration during a fall is more likely to result in less head stabilization and, ultimately, a greater risk of head impact. While this investigation did not observe an age by impact interaction, it is feasible to speculate that, under higher acceleration falls, older, less fit individuals would be less able to stabilize their head, which results in greater head impacts and an increased risk of TBIs. More research is needed to better understand the causal relationship between head acceleration and head impact as it relates to fall-related TBIs.

The disproportionate and significant adverse consequences of fall-related TBI are clear motivation for innovative prevention strategies [1–3]. It remains to be seen if interventions targeting head control can reduce fall-related TBI risk. To maximize success, preventative strategies in older adults should target modifiable risk factors. A potentially modifiable risk factor in the prevention of fall-related TBI may be neck muscle strength and activation. Resistance training for the neck muscles has been shown to effectively decrease linear head velocity during head loading conditions in young adults [33]. Conversely it has been suggested that dynamic neuromuscular training may provide more benefit for the neck musculature's response to perturbation than resistance training alone [34]. It is important to note that most exercise and fall prevention programs designed for older adults do not target neck musculature [35, 36]. Consequently, there is a dearth of research concerning neck muscle strength and activation and fall-related TBI in older adults and several knowledge gaps remain.

Another potential strategy may be teaching older adults protective movements strategies to prevent head impact during a fall. There is some evidence to suggest the possibility that head impact can be reduced during a sideways fall by individuals rotating to land on their back [37]. Furthermore, it has been shown that utilizing safe landing strategies can reduce the impact load during a fall [5]. With these techniques, it may be potentially beneficial to add functional training of the neck to support the head during a fall to prevent head impact. Still knowledge gaps remain related to understanding how teaching older adults safe landing strategies can reduce head impact and fall-related TBIs.

While this investigation has observed novel results, some limitations should be noted. The sample size was small and the participants expected the falls. The older adult participants were relatively young, healthy, and physically fit. It is speculated that older, weaker individuals would experience even more head impacts. The falls were experimentally induced and it is not clear how well they mimic real world falls. Additionally, we utilized a video analysis to quantify head impact, instead of motion capture data; more research is needed to understand a potential acceleration threshold of head impact during a fall. Furthermore, neck strength and muscle activation was not assessed, which limits the interpretations of age-related changes to neuromuscular control being a contributing factor for increased proportion of head impacts and head acceleration. More research is needed to better understand the role the neck musculature plays in head control during a fall in older adults and the potential impact low neck strength and slow muscle activation has on head acceleration during a fall.

## 5. Conclusions

This investigation was the first to highlight that older adults experience a greater proportion of head impacts during experimentally induced sideways falls. Additionally, the falls which resulted in head impacts also resulted in greater head acceleration. Age-related neuromuscular changes may be responsible for the higher proportion of head impacts in older adults. Several knowledge gaps, subsequent research examining head acceleration during a fall, the implications of neck musculature on head acceleration in older adults, and the relationship between head acceleration and fall-related TBI are still warranted.

## Figures and Tables

**Table 1:** Participants' demographics.

	Young	Older	p-value
N	10M/5F	7M/3F	-

Age (years)	21.2±2.7	61.9±4.3	<0.001*∗*

Height (cm)	172.7±7.6	170.7±7.7	0.541

BMI (kg/m^2^)	23.9±3.5	25.8±5.0	0.294

Bone Mass Density (t-score)	0.4±0.8	0.2±0.8	0.403

5-Time Sit to Stand (sec)	6.6±1.4	7.5±1.4	0.127

MoCA Score	28.3±1.7	28.6±1.2	0.673

All values are presented as mean±SD; *∗* denotes statistically significant difference

**Table 2:** Physiological profile assessment results.

	Young	Older	p-value
Edge Contrast Sensitivity (dB)	22.5±0.9	20.3±0.9	<0.001*∗*

Lower Limb Proprioception (degrees)	1.8±0.9	2.3±1.0	0.217

Knee Extension Strength (Kg)	41.9±11.6	37.0±7.7	0.270

Finger Reaction Time (ms)	226.9±48.3	219.8±44.5	0.721

Anterior-Posterior Sway (mm)	23.5±8.9	36.8±9.5	0.002*∗*

Medial-Lateral Sway (mm)	19.1±6.7	29.3±16.4	0.054

Fall Risk Score	-0.4±0.6	0.7±0.8	0.014*∗*

All values are presented as mean±SD; fall risk score was determined by short form of the physiological profile assessment [15]; *∗* denotes statistically significant difference

## Data Availability

The data used to support the findings of this study are restricted by the University of Illinois at Urbana-Champaign Institutional Review Board in order to protect participants' confidentiality. Data are available from Jacob Sosnoff (jsosnoff@illinois.edu) for researchers who meet the criteria for access to confidential data.
